# Book Reviews

**Published:** 1831-06

**Authors:** 


### CRITICAL ANALYSES.

Quæ laudanda forent, et quæ culpanda, vicissim

Illa, prius, cretâ; mox hæc, carbone, *notamus*.—PERSIUS.

*The Effects of the principal Arts, Trades, and Professions, and of Civic States and Habits of Living, on Health and Longevity: with a particular Reference to the Trades and Manufactures of Leeds; and Suggestions for the Removal of many of the Agents which produce Disease, and shorten the Duration of Life.* By C. Turner Thackrah.—8vo. pp. 126. London: Longman and Co.

Without due exercise of the mental and bodily faculties, life cannot be enjoyed, because health cannot be possessed. It is happily true that most corporeal and mental occupations, when they are not carried to excess, contribute to the full developement of all the powers of man. The most salubrious and invigorating employment is often, however, rendered prejudicial to health by attempts to perform more labour than can be borne with impunity; while there are some peculiar circumstances, incidental to different trades, arts, and professions, which expose the artisans who are employed in them to various degrees of danger. It is, of course, of the highest importance to ascertain whether these dangers cannot be obviated, and whether, by the aid of philosophy and science, the comfort, as well as safety, of those who supply our real and imaginary wants may not be materially increased.

To the investigations of these questions, Mr. Thackrah has directed his attention, and, in the course of his inquiries, he has collected much interesting information. He has thrown out various suggestions, which, if adopted, would add much to the happiness, as well as the health, of those engaged in certain trades; and it is not unreasonable to presume that, if the investigation be continued with equal zeal and intelligence by others, great improvements may be obtained. Of all the discoveries which give renown to the name of Davy, none can take precedence of the safety lamp, by which so many valuable lives have been, and will continue to be, preserved. The miner now continues his avocations in comparative safety; and we doubt not, if the inconveniences and dangers which are connected with some other occupations are earnestly investigated, that much may yet be done to diminish them.

Mr. Thackrah commences his essay by shewing that the duration of human life is considerably less in the West Riding, the manufacturing district, than in other parts of Yorkshire. It appears, also, that, in the borough of Leeds, 450 persons die annually from the injurious effects of manufactures, the crowded state of population, and the consequent bad habits of life; and that nine tenths of the survivors suffer impaired health, lingering ailments, and premature decay both of mind and body. Such statements are more than sufficient to prove that a strict examination is demanded into the state of our manufactures, and it is the purpose of the present work to excite the public attention to the subject. The manner in which Mr. Thackrah has collected his materials is thus stated.

"Myself and my pupils have personally and carefully inspected the state of the artisans in most kinds of manufacture, examined the agencies believed to be injurious, conversed on the subject with masters, overlookers, and the more intelligent workmen, and obtained many tables illustrating the character of the disorders prevalent in the several kinds of employ. From these sources collectively, I have drawn up statements, which, though avowedly imperfect, must, I conceive, approach to the truth. It will be remembered that the subject is new; that the West Riding manufactures have not before been examined in their important relations to health and longevity, and that scarcely any thing has been published even on the employments common to England at large. I have had therefore to enter a new track, without guide or assistance." (P. 5.)

For the convenience of inquiry, Mr. T. divides the inhabitants of Leeds, and its immediate neighbourhood, into four great classes: 1, Operatives; 2, Dealers; 3, Master Manufacturers and Merchants; 4, Professional Men. In examining the state of these severally, our attention is directed to the atmosphere they breathe, the muscular exercise they take, the postures of body they maintain, the variations of temperature and humidity to which they are exposed, their diet and habits of life, and, finally, in some cases, the state of the mind. He begins with those operatives who approach nearest to the perfection of the physical state: they are men of active habits, and whose employments are chiefly in the open air.

"*Butchers* stand at the head of this division. They are much in the open air, and take strong exercise. Most of the masters ride on horseback to the neighbouring markets, and often traverse the surrounding country to buy cattle. They are well known to ride fast, and to take often long journies.

*Drovers of Cattle* for the butchers, though their action is generally less violent, have great distances to travel. They walk twenty, thirty, or forty miles a day. Butchers, and the slaughter-men, their wives, and their errand boys, almost all eat fresh-cooked meat, at least twice a day. They are plump and rosy. They are generally also cheerful and good-natured. Neither does their bloody occupation, nor their beef-eating, render them savage, as some theorists pretend, and even as the English law presumes. They are not subject to such anxieties as the fluctuations of other trades produce; for meat is always in request, and butchers live comfortably in times as well of general distress as of general prosperity. They are subject to few ailments, and these the result of plethora.

The atmosphere of the slaughterhouse, though sufficiently disgusting to the nose, does not appear to be at all injurious to health. The mere odours of animal substances, whether fresh or putrid, are not apparently hurtful; indeed, they seem to be often decidedly useful.* Consumption is remarkably rare among the men employed in the slaughterhouse. If we see a phthisical youth in the fraternity, we shall generally find that his parents, aware of an hereditary disposition to consumption, brought him up to the business with the hope of averting this formidable malady. The atmosphere of the slaughterhouse, imbued with a foreign admixture, is moreover less susceptible of those natural changes, which produce epidemics. From this circumstance, conjoined with their diet and habits of life, butchers are less subject than other trades to cholera and dysentery. To the same favorable combination, we attribute their comparative exemption from diseases, considered as infectious or contagious. Of 520 patients taken to the House of Recovery in this town, during the last year, only one was a butcher, and his was a case not of typhus, but of simple fever." (P. 8.)

Dr. Tweedie† also mentions it as a singular circumstance, that, though almost every description of mechanics was ad-

* In our Number for March 1829, p. 277, there is an interesting article on the "Innocuous Nature of Putrid Exhalations."

† Clinical Illustrations of Fever, p. 79; 1830.

mitted into the Fever Hospital, he did not recollect a single instance of a butcher being sent into the establishment. It is stated by writers on the plague, that, when this pestilence last visited London, butchers were rarely attacked. Still it appears that longevity is not greater in butchers, than in the generality of employments: Mr. Thackrah suspects that it is even shorter than amongst most other men, who spend as much time in the open air. "Butchers, in fact, live too highly: not too highly for temporary health, but too highly for long life." The ready mode of preventing the maladies to which they are commonly liable, is obvious.

*Cattle and horse dealers*, leading an active life in the open air, are generally healthy, and would be almost exempt from ordinary maladies, were it not for their habit of drinking.

*Fishmongers* are greatly exposed to the weather, but are not subject to rheumatism, or other inflammatory disease.

*Labourers in husbandry, sand loaders*, and *men employed on the roads*, obtain too scanty means of subsistence to support their bodily vigor.

*Brickmakers* have full muscular exercise in the open air, and are subject to the annoyance of cold and wet.

*Brickmakers*, half naked, and with their bare feet in the puddle all day, are not more liable to catarrh, pnuemonia, and rheumatism, than men whose work is under cover and dry. Of twenty-two brickmakers of whom we made personal inquiry, only one had been affected with rheumatism, or could state himself subject to any disease. All declare that neither rheumatism, nor any inflammatory complaint, is frequent among them. Individuals of great age are found at the employ." (P. 11.)

*Chaise drivers, coachmen*, &c. are much exposed to atmospheric vicissitudes, but Mr. Thackrah believes that rheumatism and inflammation of the lungs would rarely occur among them, provided they were abstemious.

*Postilions*, of course, have great and continued exertion; but the kind is objectionable. Their position on the saddle is bad, and they use the arms unequally; hence curvature of the spine. They are moreover said by Morgagni to be particularly subject to aneurism of the aorta. The drivers of chaises and hackney-coaches have more moderate and equal exercise; but their position subjects them to popliteal aneurism." (P. 11.)

It does not appear that *coach builders, carpenters, coopers, ropemakers*, or *paviers*, suffer any very particular inconvenience from their respective occupations.

The next division of the labouring class is that in which the employments are carried on in an atmosphere confined and impure.

"We scarcely need remark that the air of a town like Leeds is always in an unnatural state. The excess indeed of carbonic acid gas is said to be very trifling; but our skins and linen prove an abundant admixture of charcoal itself. Ammoniacal and other vapours from manufactories, sewers and places of refuse, add to the general impurity. This state of atmosphere affects in a greater or less degree all the inhabitants. The complexion is pallid; and the tongue shews that digestion is disordered and imperfect. I should think that not ten per cent. of the inhabitants of towns like Leeds enjoy full health. Were we to ask, indeed, those we see around us, the major part would say that they are quite well. But a close examination would prove that there are few individuals who have not either disease of some organ, or an evident disposition to disease.

"The lungs, however, suffer much less from the air of towns than we should expect. Bronchial affections indeed are common, but other acute diseases of the chest, as pleurisy and inflammation of the lungs, are, I think, neither so frequent nor so severe as in the agricultural districts. Cases of consumption also are not comparatively numerous; nor is their progress so rapid in smoky towns as in the purer air of the country and the mountains. I speak of the general atmosphere of towns; for we shall hereafter see that the atmosphere of certain manufactories excites consumption to a very lamentable extent.

"Though all inhabitants of large towns suffer in a greater or less degree from the impurity of the atmosphere, yet it is obvious that those who are most crowded together will be chiefly affected, particularly if ventilation be imperfect. A serious addition to the evils of a confined atmosphere is the defect of muscular exercise. Certain classes of muscles are for twelve or fourteen hours a day scarcely moved, and postures maintained injurious to the proper actions of the internal organs." (P. 14.)

*Tailors* sit all day in a confined atmosphere, and often in a room too crowded, with the legs crossed and the spine bowed; they cannot have respiration, circulation, or digestion, well performed. Their employment, the author admits, produces few acute diseases: stomach and bowel disorders are general, and often obstinate, among them; pulmonary consumption is also frequent. We see no plump and rosy tailors; none of fine form and strong muscles; the spine is generally curved.

"The position of the tailor might be amended. He now sits cross-legged on a board; because in the ordinary sitting posture he could not hold a heavy piece of cloth high enough for his eyes to direct his needle. Let a hole be made in the board, of the circumference of his body, and let his seat be placed below it. The eyes and the hands will then be sufficiently near his work; his spine will not be unnaturally bent, and his chest and abdomen will be free.

I am aware that old workmen will be unwilling to regard this or similar suggestions: for every man is formed to his habits. If however masters and medical men would urge an alteration, and if especially boys apprenticed to the trade were taught to work in the posture recommended, tailors would assuredly become much more healthy. The practice of drinking might also be easily reduced, if masters discharged from their employ every man who absented himself a day without proper cause." (P. 18.)

*Milliners, dress-makers, and straw-bonnet makers,* are often crowded together in small apartments, and kept at work an improper length of time.

"In stoving strawbonnets, sulphur is largely used. The fumes, in some houses, spread through every apartment, and the inmates even sleep in an atmosphere impregnated with these offensive vapours! Sulphurous gas, I need scarcely add, greatly affects respiration. It induces at the time a violent cough, and the irritation, if frequently repeated, tends to the developement of pulmonic disease. Might not the sulphurous fumes be absorbed or confined in the process? Water in a large shallow dish would take up a considerable proportion. A small outbuilding for the operation would be a more decisive remedy. This indeed is used by some straw-bonnet makers.

"Remedies for the other evils to which this class is exposed, are obvious: ventilation, reduction of the hours of work, and brisk exercise in the open air. The great cause of the ill-health of females who make ladies' dresses, is the lowness of their wages. To obtain a livelihood, they are obliged to work in excess." (P. 19.)

We pass over the brief yet interesting sketch which the author gives of the various other occupations of this division, in but a few of which the health appears to suffer any material injury.

The next employments which are examined are those which produce dust, odour, or gaseous exhalations: they are divided into those in which the vapour, odour, or dust, is not apparently noxious; those in which it appears to be even beneficial, generally or partially; and those in which it is decidedly injurious.

We select the account which Mr. Thackrah gives of those trades in this division which seem to exert a decided influence on the health of the workman.

"*Tanners,* it is well known, are subject to disagreeable odours: they work in an atmosphere largely impregnated with the vapour of putrifying skins, and this combined with the smell of lime in one place, and of tan in another. They are exposed constantly to wet and cold; their feet are scarcely ever dry. Yet they are remarkably robust; the countenance florid; and disease almost unknown. Tanners are said to be exempt from consumption; and the subject has of late been repeatedly discussed in one of the medical societies of London.* We have carefully inquired at several tan-yards, and could not hear of a single example of this formidable disease. We do not find *old* men actually in the employ; and the reason assigned is, not the decline of health, but the inferiority of men past middle age, in undergoing the labour of the process. Persons however in advanced life, yet healthy, are found in other occupations, who have before been for many years in the tan-yards, and have not apparently suffered from the long continued exposure to their offensive odour. Hence we may infer that this employ, while it invigorates the constitution in youth and middle age, does not sensibly shorten life; does not, in other words, give temporary health at the expense of premature decline." (P. 35.)

Ramazzini states that, at Padua, the tan-yards were permitted only in the suburbs. As a matter of medical police, however, the author sees no occasion for their exclusion from the town.

*Corn millers,* breathing an atmosphere loaded with particles of flour, suffer considerably: they are generally pale and sickly; most have the appetite defective, or labour under indigestion; many are annoyed with morning cough and expectoration; and some are asthmatic at an early age.

"The evils of the employ might be much reduced by the men's taking exercise in the open air. It is apparent that working from twelve to twelve, they have time to enjoy a pure atmosphere for several hours a day. In this, as well as other employments, we remark with regret the men's inattention to health, their indifference to the prevention of disease. They think nothing of injurious agents till their health is destroyed, and the time for prevention is past. The dust might, I conceive, be removed, or greatly diminished, by a current of air under the floor. The ill effects on hearing, of this and other noisy occupations, might be diminished or prevented by putting cotton in the ear passages." (P. 37.)

*Maltsters* are exposed to much dust, to great heat, and to sulphureous fumes from the coke: they are frequently affected with bronchial inflammation, and many become asthmatic for life. The exertion is so great, that it obliges some to leave the employ at an early age, and it is much too severe for the old: hence we find no labouring maltster advanced in years.

*Workers in flax,* from their number and the effect of their employ, deserve particular attention. Dust is produced in nearly all the departments of the flax-mills: the worst department is the heckling.

* Dr. Dods brought this subject before the Westminster Medical Society: his "Essay on the Exemption of Operative Tanners from Pulmonary Consumption," is published in the Medical Gazette, vol. iii. p. 497.—Editor.

"This, in some mills, is carried on by hand, and in such the rooms are greatly clouded. In other mills, where the process is effected by machinery, the quantity of dust is considerably less. Still, however, it is such that a visiter cannot remain many minutes without being sensible of its effects on respiration. Children and a few overlookers are here the operatives; but in the old mode, I believe, men only are employed. Though attention is generally paid to ventilation, and the rooms for the several departments are spacious, they are not sufficiently lofty. A suffocating sensation is also often produced by the tubes which convey steam for heating the rooms. Persons in the dusty departments are generally unhealthy. They are subject to indigestion, morning vomiting, chronic inflammation of the bronchial membrane, inflammation of the lungs, and pulmonary consumption. The dust, largely inhaled in respiration, irritates the air-tube, produces at length organic disease of its membrane, or of the lungs themselves, and often excites the developement of tubercles in constitutions predisposed to consumption. There is little doubt that a considerable quantity is also swallowed with the saliva, and deranges, in a greater or less degree, the functions of the stomach.

"The majority of operatives in the great flax-mills are young women, girls, and boys. In twenty-three of these taken indiscriminately as they came from the mills, we found the air exhaled at an effort, to average six pints in males, whose ages averaged eighteen years; and three and five twelfths in females, whose ages averaged nineteen years. The younger operatives, who are generally of the age of from seven to twelve, were not examined.

"As the stethoscope could not be satisfactorily used in the place, and I wished to examine the health of such as have worked in the dusty departments for an unusually long period, and still continue the employ, I requested a few such individuals to be sent to my house for inspection. Six came; and in each I found the lungs or air-tube considerably diseased." (P. 40.)

The cases of the persons here referred to are briefly detailed. The process of heckling flax is, generally, Mr. Thackrah says, most injurious to health. A large proportion of men in this department die young: very few can bear it for thirty years, and not one instance could he find of any individual who had been forty years either in this or any of the dusty rooms.

"We find, indeed, comparatively few old persons in any of the departments of the flax-mills. On inquiry at one of the largest establishments in this neighbourhood, we found that of 1079 persons employed, there are only nine who have attained the age of fifty; and besides these, only twenty-two who have reached even forty." (P. 43.)

The custom of employing young children in the mills is, very properly, severely deprecated as shocking to humanity. The time of labour in the flax-mills is excessive, but we are informed that, so established are the hours of work, that no individual master can, without loss, liberate his people at an earlier period.

"A legislative enactment is the only remedy for this, as well as the other great opprobrium of our manufactures. Were a Bill drawn up to limit the duration of labour, and prevent the improper employment of children, I feel assured that it would be well supported by petitions, not only from the public, but from the masters themselves.

"The other evils of flax mills more directly destructive to health, dust, and accidents, the masters have endeavoured to diminish. In the rooms where tow is prepared the machines are covered by boxes, which collect a large quantity of the dust; and the new machines for roving the tow produce less dust than the old ones. Accidents too are rendered much less frequent, in all the departments, by the casings of the wheels. But although something has been done to save the workmen from the injurious effects of their occupation, more remains to be done.

"May I suggest a plan for carrying off the dust? Let channels, about a foot in breadth, be made in the floors, each with one end opening into the room, and the other outside of the building. Over the former let a light broad wheel, attached to the machinery, be made to revolve rapidly. A current of air will thus be produced, and this entering the channel, will draw down the greater part of the dust, and carry it out of the building. If the plan succeed in the flax mills, it would avail also for removing the dust of corn and malt mills, indeed of all the manufactures which affect the lungs by mechanical irritation. A subject of such great importance to health and longevity will receive, I trust, the attention of those who are not only much more conversant than I, with contrivance and invention, but more directly obligated by social principle to improve the state of the operatives, by whose labours they are enriched." (P. 46.)

*Masons* inhale particles of sand and dust, which arise from chipping the stone: they are dissipated in their habits, are short-lived, and generally die before the age of forty.

Mr. Thackrah introduces some interesting remarks on the state of *miners*, though he has not had the opportunity of personally examining their occupation.

Draw-filing cast iron is a very injurious occupation: the dust is much more abundant, and the metallic particles much more minute, than in the filing of *wrought* iron. The filers are almost all unhealthy men, and remarkably short-lived. Magnetic mouth-pieces, which attract the particles of iron inhaled in respiration, and thus greatly diminish the quantity which would enter the air-tube, were many years ago introduced in Sheffield, and ought, says Mr. T., ere this to have been tried at Leeds: but it appears that both men and masters are strangely indifferent to a subject which, it might be presumed, would excite their closest attention, and the exertion of all their ingenuity. As the working of wrought iron is found to be much less injurious to health than that of the cast, it is natural to ask, "Could wrought iron be used for all purposes? It is well known to be most suitable for common implements. Would it serve for large wheels, cannon, and the like? Does the comparative softness of this substance present an objection?" It is presumed that the expense is the great obstacle.

In speaking of the injurious effects which arise from the occupation of *plumbers*, the author suggests an inquiry which should not be forgotten by the intelligent and philanthropic chemist; namely, "Is there no substance which, being volatilised at the same time, would combine with the oxide of lead, so as to produce a less injurious compound?"

After having adverted to the principal occupations which affect health by the noxious substances which they offer to respiration, the next referred to are those which injure or annoy by acting on the skin.

"*Potters* suffer from the lead used in 'glazing.' Immersing their hands in a strong solution of this mineral, they are often attacked by colic; and if kept in this department, they at length become paralytic. Potters are remarkably subject to constipation of the bowels. Of seven individuals taken indifferently, we found five affected with this complaint.* The intemperate men, however, are those who chiefly suffer from the employ. In the Leeds Pottery we remarked nothing injurious but the department of glazing.

"Could not the process be effected without the immersion of the hands in the metallic solution? Or could it not be effected by a machine? Or could not some article less noxious be substituted for the lead? On visiting the Derby Pottery, some years ago, I learnt that little lead is used in the composition for glazing, and that the workmen consequently are not injured."† (P. 58.)

*Hatters, grocers,* and *chimney-sweepers,* are subject, from their employment, to affections of the skin. Almost all the occupations of a manufacturing town render the skin foul,

* One of these had been without an evacuation for six days, another for eight, and a third for eleven.

† "In an extensive lead factory in the vicinity of the metropolis, in which the colic peculiar to such places was formerly very prevalent, that disease has become so rare, that medical assistance has not, for some years past, been required. Many have supposed that the fumes of the lead induced the disease; but the remedy was found by tracing the cause to a more direct source.

and thus, of course, obstruct the important functions which this organ is destined to perform. Careful habits of cleanliness, which at present are so much neglected, would doubtless materially remedy the mischiefs which arise from this source.

Mr. Thackrah mentions an important fact connected with the manufacture of wool.

"This article is so moistened with oil, that the exposed skin of the workmen is always greasy. The effect, if we can speak of it separately from other circumstances which act on the health, is decidedly good. The men, the young women, and children in this employ, are more robust than other artisans; and when the dye and dirt are removed from the skin, have really the complexion of health. Individuals too, and especially children, who have been injured by the dust of other kinds of manufacture, and hence have been obliged to leave such employ, become hale and vigorous on their removal to the woollen. I would not, however, be understood to attribute the improvement solely, or even chiefly, to the application of oil to the skin: this article has a more important effect in preventing the formation of dust. Yet still, when we compare the state and appearance of workmen in other manufactures, where the dust is trifling, and other circumstances nearly equal, if we compare these men with the plump and rosy Slubbers, we cannot but ascribe a beneficial agency to the oily state of the skin. The subject is of practical importance." (P. 62.)

The condition of those operatives who are much exposed to wet and steam, is next considered, but we can only give the conclusions which the author has arrived at upon these subjects. They are decidedly opposed to the current opinions upon the subject, but nevertheless we believe them to be well founded. Mr. Thackrah refers to the situation and employment of several classes of society, as brickmakers, bricklayers, papermakers, &c., for the purpose of shewing that wet and cold, without other agencies, do not produce the disorders ascribed to them.

"The inferences, then, from our examination of particular employments and classes of men, as well as those deduced from general practice, are 1st, that 'wet and cold,' as they occur in ordinary life, are rarely adequate to the production of disease; and 2d, that, in the few cases in which they have such agency, they are only the exciting causes of disease." (P. 69.)

Workmen are seldom very strict in regard to cleanliness. The probability of particles of the mineral being conveyed from the hands amongst the food was suggested, and an order enforced that before any of the workmen should leave the factory to go to meals, their hands should be thoroughly washed, and that nail-brushes should be used to prevent any of the lead remaining where it was most likely to adhere. The success of this plan, under strict superintendence, has been complete."—(Alcock on the Education of the General Practitioner.)

The health of men exposed to a high temperature, or to great variations of temperature, is next investigated. The author is led, from his observations, to conclude that habit has little power in rendering the body insensible to heat. Operatives habituated to high temperature daily feel effects similar to those felt by persons who only occasionally place themselves in this temperature. The men daily have an excitement of pulse, perspiration proportioned to the degree and continuance of the heat, and its complication with muscular labour, thirst, and languor; the complexion is rendered pale, and the digestive functions are impaired. The remedies suggested for the evils referred to in this section are

"1. Diminution of the muscular labour which is performed in hot rooms. Raising the iron tenter-frames in the dry-house ought to be effected, and the hot plates of the stuff-pressers conveyed, by machinery. These, and similar modes of relief, are more worthy of mechanic ingenuity, than most of the ends to which this ingenuity is devoted. The men, moreover, should be less active, and carry lighter weights. In other countries, heat is considered a sufficient cause for the reduction of labour; while in England, operatives employ all their strength, as well in a temperature equal to that of the tropics, as in the open air of our winters. 2. The drinking lemonade, or other diluent, during the time of labour, rather than the noxious compound called ale. 3. The use of stimulants with the food, after labour. 4. The reduction of the period of labour." (P. 80.)

The influence of their respective employments upon the health of *shopkeepers, commercial travellers, merchants,* and *master manufacturers,* is next considered. We can extract but one passage, which conveys a very important lesson, although we fear that the *scabies lucri* has too firm a hold upon the minds of the mercantile classes of society, to induce them to lose a little gain, even for the obtainment of that much more valuable possession, health.

"The physical evils of commercial life would be considerably reduced, if men reflected that the success of business may be prevented by the very means used to promote it. Excessive application and anxiety, by disordering the animal economy, weaken the mental powers. Our opinions are affected by states of the body, and our judgment often perverted. If a clear head be required in commercial transactions, a healthy state of the body is of the first importance; and a healthy state of body is incompatible with excessive application of mind, the want of exercise and of fresh air. But subjects like this find no entry in the books of our merchants. Intent on their avocations, they strangely overlook the means necessary for pursuing them with success. They find, too late, that they have sacrificed the body to the mind." (P. 85.)

*Professional men*, and *persons engaged in literature*, form the last class for examination. The author then gives a recapitulation, or abstract, of the effects of the principal employments of large towns on the health of those employed; and concludes the volume with several judicious suggestions, which, if carried into effect, would much tend to add to the happiness and health of the operative classes of society, in whose welfare all must be deeply interested.

We have now given a general sketch of Mr. Thackrah's work, and we feel assured that, whoever peruses it, will feel grateful to him for the valuable information he has collected upon the very important subjects to which he has directed his attention.

---

*The Pharmacopœia Universalis; or, Complete Encyclopædia of the Materia Medica, contained in the Pharmacopœias of London, Edinburgh, and Dublin, as well as of all those of Europe and America; and of the Dispensatories, Formularies, and Chemical Works of Ainslie, Bigelow, Brande, Brera, Brugnatelli, Buchner, Chevalier, Chevreuil, Coxe, Cullen, Davy, De Lens, Duncan, Gray, Guibourt, Henry, Hufeland, Magendie, Murray, Orfila, Paris, Phillips, Piderit, Poiret, Ratier, Richard, Robinet, Rose, Spielmann, Thomson, Van Mons, and numerous others: giving the Officinal and other Synonymes and Scientific Characters of Simple Substances, the Preparations of the Compounds, according to the various Formulæ, and the usual Doses prescribed.* By A. J. L. Jourdan, M.D., of Paris.

*With an Appendix, containing the Charters, Laws, and Regulations, affecting the Medical Profession; and an Outline of Medical Chemistry, and Manipulations in the Manufacture of Drugs and Medicinal Waters; including the most recent Discoveries in Pharmacy.* Edited by J. Rennie, A.M. A.L.S. &c. &c.

Part I.—8vo. pp. 96. 1831.

The titlepage of this work so fully proclaims its objects, and announces its contents, that little remains for us to do but to illustrate the mode in which the multifarious task has been performed in this First Part, which promises well for its successors, if there be any truth in the universal proverb, "Pares cum paribus facillime congregantur:" we, however, with the characteristic caution of the North, though anxious to bestow praise where praise is due, not being, as some of our brethren are, endowed with the privilege of second-sight, withhold our final opinion until we have seen the work completed; well knowing that, on the authority of Aristotle, another proverb says, "Τὸ ἔαρ μιά χελιδων ὀυ ποιεῖ."

We love simplicity, and hence have viewed with pleasure the London College of Physicians curtail, in each succeeding edition of its Pharmacopœia, the catalogue of drugs, which, from their former multiplicity, could seldom be expected, if herbs, to be fresh, or in a fit state for medicinal administration: still, though we think the national code should be as simple as possible, it is obviously necessary that some more comprehensive work should be within general reach, to which reference can easily be made, as to the substances and preparations used and recommended in continental and other foreign Pharmacopœiæ, and a comparison be instituted as to the advantages of the different processes which the different authorities severally recommend. Now this desideratum promises to be given in the publication of the "Pharmacopœia Universalis;" and, in our opinion, Mr. Rennie is doing a service to science in thus bringing Jourdan's work prominently before the English student.

We subjoin two specimens.

"Acidum Hydrocyanicum. Prussic Acid, Hydrocyanic Acid.

"*Acidum Borussicum*, seu *zooticum*, seu *zootinicum*. *Acidum Prussicum*. (Dubl.; Amer.; Belg.; Batav.; Gall.; Ferr.; Niemann; Brugnatelli; Van Mons.)

"1. Prepared after the method of Scheele.

℞. Prussian Blue, one hundred and twenty-eight parts.
Red Oxide of Mercury, sixty-four parts.
Distilled Water, five hundred parts.

Boil for a quarter of an hour, stirring constantly; strain, filter, and wash the residuum with

Boiling Water, one hundred and twenty-eight parts.

Mix together the two liquors, put them into a flax, and add

Iron filings, reduced to very fine powder, ninety-six parts.
Sulphuric Acid (66 degrees), twenty-four parts.

Diluted with

Distilled Water, twenty-four parts.

"Stir the mixture, and keep the flask, for one hour, plunged in cold water; pour the decanted liquor into a tubulated retort placed in a sand bath, to the neck of which a long adapter is fixed, which passes into the tube of a globular receiver, whence goes out another tube, of a bent form, which is plunged into a flask full of water; lute the apparatus, cover the receiver with wet cloths, and increase the heat until the liquor boils, and it has passed

One hundred and ninety-two parts of liquid into the receiver.

Add to this liquid,

Subcarbonate of Lime, eight parts.

Distil again, and draw one hundred and twenty-eight parts.

Preserve in a flask covered with black paper. (Amer. Gall.; Ferr.; Brugnatelli.)

"Van Mons directs three parts of cyanuret of mercury to be dissolved in water; to put the solution into a retort containing three parts of iron filings; to pour upon the whole ten parts of sulphuric acid, diluted with thrice its weight of water; to stir until the mercury is separated; to place the retort in a sand bath; to heat it to ebullition; to distil over one hundred and seventeen parts of liquid: and to rectify it by a new distillation.

"This process gives an acid, which is always mixed with water, in an uncertain quantity.

"2. After the process of Gay-Lussac.

℞. Cyanuret of Mercury, one ounce.
Muriatic Acid, seven fluid ounces.
Water, eight fluid ounces.

Distil from a glass retort, into a receiver kept cool, eight fluid ounces; and preserve in a well-stopped bottle, in a cool, dark place.

"The specific gravity of this should be .998. (Dubl.)

"℞. Cyanuret of Mercury, any quantity.

Put into a tubulated retort, the neck of which is furnished with a large glass tube, filled with marble bruised, and melted chloruret of calcium, which tube communicates by another more narrow, with a bell-glass surrounded with a refrigerating mixture. Pour upon it enough of hydrochloric acid to rise above the cyanuret the height of a finger, heat gradually and moderately, and receive the condensed product in the bell-glass. (Gall.; Ferr.; Magendie.)

"The acid thus obtained is free from water: it has a specific gravity of 0.700.

"3. After the process of Gea Pessina.

℞. Hydroferrocyanate of Potass, pulverised, eighteen parts.

Put it into a tubulated glass retort, placed on the iron grate which supports a stove, and communicating with a very small tubulated flask, the tube of which plunges into another flask containing a little distilled water; and pour upon it a mixture of

Concentrated Sulphuric Acid, nine parts.
Water, twelve parts.

Let them act upon each other for twelve hours, during which time the ice ought to be renewed, as fast as it melts, and the retort be gently heated by means of some burning coals; the fire is to be removed when a blue matter rises, and threatens to pass into the receiver, and the apparatus suffered to cool. (Henri.)

"The acid so obtained has a density equal to 0.9 or 0.898.

"4. After the manner of Vauquelin.

℞. Cyanuret of Mercury, one part.
Distilled Water, eight parts.

Pass a current of sulphuric acid gas into the solution, until the gas is in excess; then pour into the liquor as much pulverised subcarbonate of lead as will remove the excess of the hydrosulphuric acid, constantly stirring the mixture: when it no longer has the odour of rotten eggs, and it no longer blackens paper impregnated with the acetate of lead, filter, and preserve it for use. (Batav.; Belg.; Gall.; Niemann; Van Mons.)

"The acid produced by this operation has the same density as the acid of Scheele.

"The variable density of the hydrocyanic acid prepared according to the method of Scheele, does not permit it to be applied to the purposes of medicine. For such purposes, that procured by the method of Gay Lussac is generally made use of; but, as its concentration renders it dangerous, it ought to be commenced with by mixing it with a quantity of some distilled water. Robiquet has proposed to reduce its density to 0.900., by adding to it two parts of water: thus reduced, it becomes similar to the acid of Scheele, but with the advantage over the latter of exhibiting a constant and well-known proportion between the pure anhydrous acid and the quantity of water with which it is mixed. Magendie adds to it six times its volume, or eight times and a half of its weight of distilled water, and calls the mixture *Medicinal Prussic Acid*, (*Acide Prussique Medicinal.*) Others have recommended the use of a mixture of three fourths of water, and one fourth of acid, under the name of *Acide Hydrocyanique au quart.* The formula of Magendie ought to be preserved, because it is generally adopted, but for this only reason, for it has no real advantage over the others.

"Pure hydrocyanic acid is a formidable poison, as it would kill the most robust man, with the rapidity of lightning, at the dose of a single drop. Mixed with water, it has a less energetic action, the result of which is to destroy the excess of irritability which may be developed in one particular part of the body. It has been recommended in nervous and chronic coughs, asthma, hooping cough, pulmonary consumption, indigestion with or without vomiting, painter's colic, &c. Externally, it has been employed in lotions, for various cutaneous diseases, particularly for allaying the itching of the skin.

"*Alcoholized Hydrocyanic Acid.*

"*Acidum Borussicum* seu *Hydrocyanicum alcoholisatum.* (Batav.; Bavar.; Niemann; Van Mons.)

℞. Hydroferrocyanate of Potass, four parts.
Water, sixteen parts.

Add a mixture, very cold, of

Concentrated Sulphuric Acid, three parts.
Alcohol, twelve parts.

Digest with a gentle heat, stirring often; pour off the clear liquor, and distil in a retort, until the product occupies twenty times the volume of one part of water. (Bavar.)

"The specific gravity of this product is of 0.900.

"℞. Prussian Blue, thirteen parts.

Put it into a retort to which there is a rather spacious receiver adapted, and pour upon it a mixture of

Sulphuric Acid, two parts.
Proof Spirit, fifty-two parts.

Distil with a gentle heat three fourths of the spirit employed. (Van Mons.)

"℞. Concentrated Sulphuric Acid, } of each four parts.
Water, }
Prussian Blue, eight parts.
Alcohol, seven parts.

Distil. (Niemann.)

"This process is by Keller; it gives an acid, of which the specific gravity is 0.800.

"℞. Hydroferrocyanate of Potass, in powder, four ounces.

Pour upon it, in a retort, a mixture of

Concentrated Sulphuric Acid, two ounces.
Water, four ounces.

Distil almost to dryness into a receiver, containing

Highly rectified Alcohol, eight ounces.

Digest the product without heat, for some hours, with one drachm of calcined magnesia, and distil six ounces of it into a receiver containing two ounces of rectified alcohol. (Niemann.)

"This is the process of Ittner.

"Caillot has modified the process of Vauquelin, by mixing the product with four parts of alcohol at forty degrees. Magendie allows his medicinal prussic acid to be made of six times its volume of alcohol, instead of water, to the acid of Gay-Lussac. Rust has also proposed to dissolve eight drops of hydrocyanic acid in two drachms of rectified alcohol. These extemporaneous formula are preferable to the preceding.

"The addition of alcohol makes the acid preserve better its active properties, and prevents its evaporating so readily as when the mixture is made with water.

"*Hydrocyanic Acid of Harles.*

"*Acidum Hydrocyanicum dilutum spirituosum aquosum.* (Niemann.)

'℞. Hydrocyanic Acid of Keller, ten parts.

Proof Spirit, } of each, sixty drops.

Limetree Water, }

"Dose, five drops to young people, from seven to twelve to adults, and two or three to infants of seven years, to be taken in a spoonful of water. The limetree water may be replaced by rose water, and the proof spirit by spirituous cinnamon water." (P. 57,)

Then follow numerous formulæ in which this acid may be conveniently administered, as recommended by MAGENDIE, BRERA, BORIES, ELLIS, PIERQUIN, FEE, DE GASSICOURT, NIEMANN, &c., and an account of the various preparations in which it is derived immediately from the vegetable kingdom, as cherry laurel water, black cherry water, oil of bitter almonds, &c.

"ÆSCULUS. HORSE CHESNUT.

" *Æsculus Hippocastanum,* LINN.

"Synonymes: Marronier d'Inde, FR.; Rosskastanie, GERM.; Hestekastanier, DAN.; Esculo, Castana de Caballo, HISP.; Paardenkastanie, DUT.; Castagno d'India, Ippocastano, ITAL. and PORT.; Kasztan owdzikich, POL.; Hæstkastanie, Swed.

"Dubl.; Austr.; Amst.; Batav.; Bavar.; Belg.; Bruns.; Dan.; Gall.; Ferr.; Fuld.; Genev.; Hann.; Lipp.; Olden.; Pol.; Borus.; Ross.; Sax.; Suec.; Wirtem.; Herbip.; Brugnatelli; Coxe; Guibourt; Murray and Gmelin; Spielmann.

"This tree is common in Asia. (*Heptandria Monogynia,* LINN.; *Acerideæ,* JUSS.; *Fig. Nouv. Duh.* ii. 13, 14.)

"The bark (*Cortex Hippocastani* seu *Castaneæ equinæ*) is light, of the thickness of two or three lines, and brittle; grey, or of a reddish brown colour, without; yellow, pale, or iron coloured, within. It has a slightly aromatic odour, and its taste is very astringent, rather bitter, and not disagreeable.

" It is a considerably powerful astringent, and has been recommended in intermittent fevers. The dose of the powder is from two to four scruples every three hours, in the cold stage, until an ounce and a half shall be consumed.

"The seed, called *Castanea equina,* has the size, the form, and appearance of a fine chesnut, but its taste is very bitter and disagreeable.

" It contains a great deal of starch. It has been much praised, when torrified, as a remedy in atonic uterine hemorrhagies: for this purpose it is pulverised, and an ounce and a half of it is boiled with six ounces of water, till reduced to three, which the patient takes in two doses, one before, and the other after dinner.

" *Starch of the Horse Chesnut. Fæcula Fructuum Hippocastani.* (Gall.)

" ℞. Horse Chesnuts, any quantity.

Remove the skins, and rasp them: put the pulp into a linen bag, and submit it to the press; add a little water to the juice, and let it remain at rest; then pour off the clear liquor, dry the fæcula with a gentle heat, and pulverize it.

"*Factitious Powder of Cinchona.* (Niemann, Hufeland.)

| | | |
|---|---|---|
| " ℞. | Bark of Horse Chesnut, ——— Willow, | of each, half an ounce. |
| | Root of Gentian, ——— Sweet Flag, ——— Avens, | of each, two drachms. |

Reduce them to a very fine powder.

"Hufeland assures us that this powder has precisely the same effect as the cinchona, three times out of four.

"*Factitious Decoction of Cinchona.* (Spielmann.)

| | | |
|---|---|---|
| " ℞. | Bark of Willow, ——— Horse Chesnut, | of each, half an ounce. |
| | Root of Sweet Flag, ——— Avens, | of each, two drachms. |
| | Water, | sixteen ounces. |

Reduce to eight ounces, by boiling," (P. 89.)

Then follow, as before, various formulæ, which experience has proved to be the most advantageous and convenient modes of administration; so that, as the title states, there is here presented an "Encyclopædia of Materia Medica."

If the editor should give at the end of the work an alphabetical index of the French, German, and Italian synonymes, he will very materially assist the student in his perusal of professional works in those languages. We know from experience, that the chief difficulty which is felt in reading foreign medical writers, even by those who are well acquainted with their language, is to comprehend the names of different articles of the materia medica. The common dictionaries afford, in general, no assistance upon these points, and we know of no concise book of reference to which the student can resort when he finds himself in doubt.

---

*Distinction without Separation: in a Letter to the President of the College of Surgeons, on the present State of the Profession.* By Joseph Henry Green, F.R.S. F.G.S. &c. —Hurst, London.

Nothing is at present more fashionable, more misapplied, more seductive, and less understood, than the word Reform. Where *change* is uncalled for in public institutions, reform not unfrequently becomes the watchword of a turbulent and mischievous spirit of causeless discontent: when legitimately required to correct serious abuses, it affords an unanswerable argument against suffering those things which are not in just proportion to existing circumstances, to remain *as they are*. That *individual* reform is a consummation devoutly to be wished, every individual must conscientiously allow; that political reform is required, the enthusiastic movements of the day loudly proclaim; and that reform is wanted in the constitution of the medical department, both as respects the public welfare and the peculiar interests of the healing art, should also appear, by the variety of conflicting appeals on this interesting topic.

When the acknowledged learning and professional eminence of the chiefs of the existing Colleges of Medicine and Surgery are considered, it is difficult to determine the precise nature of the reform which can safely promise to improve the objects of these scientific institutions. Should it indeed be perceived, upon a strict inquiry, that the charters which preserve the privileges of the *governing* body too feebly empower them to extend protection to the public and their own members; not investing the rulers with authority to arrest the reckless career of ignorance and imposture, nor affording them the means of patronizing ability and zeal: should such be the case, no question can be raised on the necessity of reform.

If, for example, the College of Surgeons does not possess the authority to punish ignorant intruders, however zealous and skilful may be the individuals who compose the governing body, yet if they are merely clothed with the power to tax and control their fraternity, of what use is their Charter and Bye-laws to the general body of the members? Hence the appeal for a reform is reasonable. The *governed* must ever be discontented with their rulers, when they do not receive a suitable return for the price they have paid for a protection which they will always assume an undoubted right to demand. But if every quack and ignorant pretender may practise in defiance of the authority of the College, then the members at large are unprotected, and they have made their payments and submission in vain.

Should also the benevolent desires of the College of Physicians to guard the public health from ignorance and empiricism be confined within the boundary of a few miles round the metropolis, how inefficient to its salutary purpose must be the protection afforded by a Charter, which, in these circumstances, can do little beyond securing the peculiar rights and immunities of the governing body. The laws of the College, thus restricted, are a dead letter out of the line of a small magic circle; so that the rest of the empire must severely suffer by this restriction of wholesome authority: for, although some learned graduates, the fellows or licentiates of the London College, may spread themselves throughout the land, yet these must be few in proportion to the wants of society. Hence the health of the provincials is unavoidably committed to physicians who are deemed unworthy to prescribe for the sick of the capital; that is, to the scientific and experienced graduates of other Colleges.

But if these physicians are ineligible to exercise their talents in the capital, what renders them safer advisers to the sick who reside beyond the College line of demarcation?

The laws of the London College, if advantageous to the public welfare, are rendered almost useless by the limits of their effective operation; and their inadequacy, even within these limits, is confirmed by their utter inability to punish the swarm of empirics who practise under the eye of the College, with an equally destructive and disgraceful impunity: and as respects the public safety, which alone can form the ground of the utility of the collegiate code of laws in the mind of the legislature, a singular inconsistency is observable in the power given to the surgeon who obtains the certificate of the trading Company of Apothecaries, under the sanction of an Act of Parliament, to become the only physician to three fourths of the community. To say that such a medical practitioner is not a physician, and therefore does not interpose as such between the members of the London College and the precluded physicians of other schools, *because* he does not possess a medical diploma, and dare neither call himself a doctor, nor put the initials of his name at the foot of a prescription, is surely puerile trifling; unless these distinctive marks are more characteristic of his calling than are the important duties he performs to those who solicit his professional aid. Nor can it escape observation, that while the College laws effectually prevent the learned physicians of other schools from exercising their talents within London and its environs, yet the eminent surgeon, without any other diploma than that of his own College, often acquires more fame and emolument by his able advice in general disease, than by his purely surgical practice. Who wrote better, gave better advice, or took more fees, for treating diseases of the digestive and all other organs of the human body, than the late Mr. ABERNETHY? and there are many other stars of the same lustre and order.

If, then, these anomalies and inconsistencies do exist in utter defiance of the London College, or are, as a part of its system, rather permitted than openly recognized, either its laws are useless, by the feeble power of their operation, or they are worse than useless, if only opposed to the talent which is continually emanating from other universities: they are not beneficial to the public; they are baneful to the best interests of science; and hence there is a reasonable ground for believing that a reform is needful towards the better regulation of the practice of the healing art, both as respects the welfare of society, the improvement of medicine, and the respectability and comfort of the members of the profession.

In what particulars such a reform should consist, becomes a consideration of serious importance.

The *authority* to *admit* to distinction in medicine and surgery, should only be limited by an ascertained measure of attainment, with corresponding proof of moral excellence; whilst the power of *exclusion* should acknowledge no other basis than ignorance and demerit. The laws of the Colleges, having alone as their foundation the public welfare and the advancement of the healing art, should be of universal application throughout the empire; should recognize competent ability from every quarter, and should thus respect the diplomas of all other universities. Without the College recognition, no one should be permitted to practise in any department of the profession: him who would dare do so, the College should possess the power to punish. If this power be wanting, reform is necessary.

The learned author of a Letter to the President of the College of Surgeons, under the title prefixed to these cursory remarks, proposes certain modifications of their Charter, respecting the value of which, perhaps, some difference of opinion may subsist.

He suggests that the government of the College should be vested in a President, a Supreme Council, and a General Council.

That the supreme Council should consist only of those who do not practise midwifery, nor dispense medicines.

That the general Council should consist of the supreme Council, and of forty additional members, twenty of whom should be under the obligation not to practise midwifery nor dispense medicines, and the remaining twenty of *general* practitioners.

Of what possible use, either to themselves or to the institution, these twenty general practitioners can be, we are unable to discern. Although *formally* associated with the general Council, they are in truth depressed under a cloud of deep disdain: nothing better than unprivileged intruders. They form a despicable minority, ineligible to the supreme Council.

A modification follows, which demands a still more grave consideration: upon this proposition hinges all the spirit of the enlightened author's contemplated improvements.

The eligibility, it is proposed, of that class of members of the general Council, under the obligation of not practising midwifery, nor dispensing medicines, should be further determined by proofs of a *longer* course of study, and of *superior capability,* evinced by *severer* examinations.

Now to our minds it appears that the great utility of a College of Medicine and Surgery consists in its *power* and *determination* to exclude incapable persons from practising in *any* department of the healing art. It is such a wholesome interdiction that should *here* form the basis of a salutary reform, and every modification.

The public health, over which the Colleges are the conservative power, must not be consigned to the *chances* of relative competency, if ability be ascertainable by the test of examinations. The professional knowledge of medical practitioners must never receive sanction under the admeasurement of comparative degrees in the attainments and capabilities of the candidate for collegiate distinction or public confidence: should his fitness not amount to the sum of ability required by a positive rule, he is insufficient to the important trust he solicits, and from this, and this cause alone, is ineligible to the honours which *knowledge* should justly possess a right to claim.

The author of "*Distinction without Separation*" proposes, however, to receive into the governing body of the College a limited number of members avowedly considered as less able than their associates. He remarks " his deploring as an evil, in the present constitution of the College, the want of sympathy between the members and the governing body;" but contends that "the general practitioner has no equitable grounds of complaint." Upon a further consideration, he observes, that "as exclusion, even where it is not reasonable, is too natural a source of dissatisfaction," the modifications to which our attention has been called are proposed as a remedy.

But upon what just grounds can the offer be made of a premium to comparative ignorance? Are the public interests to suffer by a disgraceful compromise? Is it not beneath the dignity of the College so to regard the want of sympathy between the members and the governing body, that they should condescend to invite twenty persons into the general Council, whose professional attainments are avowed to be inferior to a great majority of their associates? who, because they dispense the medicines they prescribe, must, *in consequence,* be comparatively ignorant of their other duties. Instead of allowing *such* the honour of a seat in the Council, they should not be permitted to *enter* into practice. Against the dangerous errors of comparative incompetency, the College should direct the severest penalties with which it may be armed by the law of the land. It should be remembered that the victims of these recognized, but at the same time *assumed*, less competent members of the general Council, comprehend a majority of the public, which is, and must be, attended by the general practitioner, for obvious reasons, in the present state of the professions. To *require* superior knowledge in *certain* members only, is to license inferiority in their remaining associates; a licence which neither honour nor honesty can ever tolerate. The introduction of *avowed* incompetency in knowledge, within any department of the medical profession, is treason to the public, and aims a sure, if distant, death-blow to the advancement of medical science. To permit imperfectly educated men to prey upon the public, whilst we disdain any other than a degrading communion with comparative ignorance, in any real participation of collegiate honours and advantages, appears so opposed to humanity, so derogatory to the dignity of the healing art, that the announcement of any concession so unworthy must create a strong feeling of regret in every well-ordered mind. The same proof of knowledge should be demanded of every candidate for practice; and the proof should be as perfect as can be procured by the test of examinations. This should be required of every noviciate: his further advancement in the art must entirely depend upon his peculiar ability and zeal; it cannot be brought within the present consideration, though well worthy of it: it would form the basis of a system of medical ethics.

The objection here made to the modifications suggested by the author of "Distinction without Separation," applies to the injurious principle of tolerating the *deficient* knowledge with which, it is assumed, the general practitioner must be possessed, *so long as* he practises midwifery and dispenses medicines. The author of the Letter appears, however, satisfied to allow him to practise upon the public, and even admits him to a degrading species of participation in the government of the institution. It is this dangerous permission to practice, this seeming concession to dissatisfaction, against which we deem it a duty to protest. An inquiry presents itself, however, of a different character, demanding consideration. Is this *assumption* of comparative ignorance verified by experience? and, if it can be substantiated by competent evidence, are the modifications proposed by the author of "Distinction without Separation" adequate to the correction of so momentous an evil? If they are unequal to this important purpose, surely some other reform is required,

What renders the general practitioner *necessarily* incompetent, *because* he practises midwifery and dispenses his medicines? He is, generally, with the author of "Distinction without Separation," a member of the College of Surgeons; sometimes a proscribed graduate of a distant College of Physicians; and not unfrequently has been the experienced director of our navy and army hospitals. He is the chief medical adviser of the public at large; he pays the College physicians and surgeons for the professional education he receives: if he be not properly taught, the fault is with his teachers and examiners. A man may not profit by the best education, but then the test of examination should determine his competency; and if these be not conducted to the fulfilment of this purpose, an additional reason is afforded for the urgent necessity of a reform in existing regulations; a reform not founded upon the desire to conciliate the jarring elements of the various pretensions of opposing classes of practitioners among themselves, and involving alone their petty interests, concerning which the public welfare, and the improvement of the healing art, have no concern; but having for its basis a fraternity, whose merits shall be accurately ascertained by a rigid and highly honourable scrutiny. When the candidate has substantiated his claim by the proof demanded, he should be at liberty to practise in whatever departments of the profession he might choose. The possession of the sum total of the knowledge required by the examiners constitutes his fitness: the further result of his exertions must be inevitably left to his capability and zeal, his good or ill fortune. Provided he pass through the trial of his merit with honourable distinction, it were useless to object to the quarter from whence he has derived his information, or to the peculiar nature and extent of its application in practice.

That a comparative ignorance in the general practitioner is to be legitimately assumed from the circumstance of his uniting the several branches which form the total amount of medical duty, is nowhere satisfactorily shewn, and is opposed by the test of experience and evidence of facts. The approbation now very universally expressed by patients, even those in the higher and enlightened ranks of society, of the humanity and skill of their medical attendants of this order in the profession, affords as strong a presumption of ability as the proposed modifications and College restraints imply the want of it. In the number of those general practitioners who have been so long employed, to their honour and credit, in the public service of the state, there are many whose writings manifest that the most extensive union of medical duties does not, in truth, imply an inferiority of knowledge of any one in particular. Neither does it appear that the learning and skill of such men as Mason Good, Thompson, and others, was *less in degree* when they united midwifery and pharmacy with their practice, than after they became members of the College of Physicians: nor did the same union of duties prevent Hey, Lucas, Baynton, &c. from being universally and justly considered on a par with the *very purest* surgeons of London. In a word, if the comparative ignorance thus assumed as the ground of exclusion from the proposed "*supreme Council*" does truly subsist, the excluders exhibit a serious charge of blame on their own efficiency as a governing body, whose most important duty it is to ascertain the possession of that ability in the noviciate which the welfare of society demands: and if the implied comparative ignorance, which is assigned as a reason for fixing the general practitioner to the ground, be assumed without sufficient evidence of the fact, then the proposed prohibitory law must be founded in mere and unjust prejudice; a ground which science and good morals disdain. Or is it founded on a shallow delight of *distinction,* which is nevertheless satisfied, under the anomalous aspect of communion with inequality of merit, to sustain itself against the influence of an energetic spirit of salutary reform? Or is this degrading proposition founded upon something yet more culpable than vanity?

The College, whose chief aim is the public good and the interests of science, should not be satisfied with the ascertained full amount of the noviciate's professional attainments: it should require proof of the actual possession of the elements of a liberal education; and should it not engage itself to examine the candidate's proficiency in languages, mathematics, or other branches of liberal knowledge: it should demand from the tutors satisfactory certificates of such a proficiency having been attained. Certificates of any description, which merely state the *periods* of probationary study, are worse than useless, for they lead to deception.

We shall now offer a few remarks upon one or two passages in the Letter to the President of the College of Surgeons, although they are of inferior consideration to the proposed modifications in the laws of its constitution.

The author of the Letter advocates an "*effectual co-operation*" of the physicians and surgeons; the difference in the two classes being, as he justly remarks, "*only practical;*" whilst "a division, where it is impossible to draw a boundary line," he considers "an absurdity." He would adopt a scheme of "*ordered unity*" "distinction without separation:" that is to say, a union between the physicians and surgeons, with a distinction between these and the general practitioner; while a *kind* of communion should be held out to the latter by his admission to the general Council in the manner already announced, in order to remedy the "want of sympathy" which at present subsists, and to silence "those bitter complaints and loudly trumpeted-forth grievances, which, full of sound and fury, threaten to shake to its foundation the College of Surgeons." Hitherto, however, these "bitter complaints" have been as "a tale told by an idiot, full of sound and fury, signifying nothing." Nor do we think the author of "Distinction without Separation" has ascertained their most judicious treatment, although we should hope the quiet spirit of rational reform may in due time effect a perfect cure. We fear not, however, without such effectual alteratives as must change altogether the present ill state of the constitution.

When the author of "Distinction without Separation" estimates the general practitioner of the present day with the mere apothecaries and druggists of early times, and would continue to press him to the sordid level of this *assumed* low origin, he should condescend to call to his mind the derivation of the surgeon from the honest barber, and to the low esteem in which he was held by the physicians, who took no other interest in him, he says, than "by committing him to Newgate;" a process we should lament to witness in this reforming age. In truth, the respect due to the modern surgeon, whether practising in one or more of the departments of the healing art, should not be qualified by any reference to his origin in the dawn of science, but to his present station and attainments.

The author of the Letter is not correct in stating the origin of the general practitioner. He cannot boast of ancestry of so early a date; he is a creature arising out of the necessity of recent times; nor does he more resemble his unjustly-reputed parent, the old apothecary, than the purest surgeon of the present day resembles the antique shaver. The *change* which has *created* in these times the general practitioner, should be the object of attention; and the happy *results* of this change, arising from the gradual improvement of general and professional knowledge, should form the ground of our appreciation of character. The endeavour to raise the character of the one by depreciating the other can no longer maintain its unjust pretensions, than while public attention shall not be directed to the consideration of the subject, and reformation slumbers.

The reader of the Letter to the President of the College of Surgeons must admire the splendid picture of the advance of chirurgical science, as contrasted with the literature of lawyers and divines. A more intimate acquaintance with the learning and inestimable labours of many modern theologians cannot fail, we should hope, to gratify the author of "Distinction without Separation," and in a short time induce him to reverse the portrait.

"It is under a strong sense of a duty which I owe to my profession," the author remarks, "that unwillingly relinquishing pursuits far more congenial to my taste, I venture to address you upon this subject of present paramount importance." For so generous a sacrifice the profession is laid under a deep obligation: as, however, the *period* of this unwilling relinquishment of more congenial pursuits, we should hope could not be of very long duration, we may be permitted to flatter ourselves that the claims of science have not sustained much serious interruption, nor the author's taste any permanent vitiation. He may surely pardon himself for appropriating ***an hour*** to fulfil a strong sense of duty on a subject of present paramount importance.

It remains for us to observe, that the author of "Distinction without Separation" seems to doubt that a minister would grant his modifications of the present Charter; and we are happy to coincide with the opinion which should favour an abandonment "of any partial alteration or regulation" for the better adoption of *one* faculty of medicine, founded on the principles of philanthropy and the promotion of science.

---

### *On the Physiology of the Fœtus, Liver, and Spleen.* By George Calvert Holland, M.D.

(Concluded from page 441.)

*The Physiology of the Fœtus.* "The various opinions entertained concerning the nourishment of the fœtus, by Hippocrates, Galen, Harvey, Darwin, Bonetus, and many others," which declare that it is jointly performed "by means of the mouth and the umbilical vessels," some believing it to be chiefly nourished by absorption, or reception into the stomach, of the liquor amnii, and some "regarding the placenta as alone necessary for the purposes of fœtal nutrition, as well as the pulmonary functions attributed to the placenta by Mayon, Bostock, and Abernethy; the galvanic hypothesis of Wilson Philip; the conjoined respiratory functions of the skin and amniotic liquor, supposed to exist by MM. Chevreuil and Lassaigne; together with the power of secreting albumen for the nourishment of the fœtus, attributed to the liver by Dr. Lee, having all been summarily adverted to, and rejected, in the first chapter, a clear field is left for the author to raise his own superstructure on the ruins of the theories of his predecessors; and this he commences with some observations on "*the primary and secondary functions of life.*"

"If we succeed in distinguishing the proper office of each organ, we shall perceive that some have a direct, and others an indirect, influence in producing one great result, viz. the nourishment of the body. The brain and the spinal cord are essential to intellect, sensibility, and motion, which are properties of animal life; but they are not indispensably necessary to the functions of organic life." (P. 49.)

And then, after citing, as illustrations, those lower animals in which certain parts are absent, as well as cases of lusus naturæ, in which they have not been formed, he thus proceeds:

"Additional organs are bestowed on the animal of more complex construction, that it may answer the varied purposes for which it was intended. What these purposes are, and how far they are accomplished, it is not my present object to describe. The brain, the spinal cord, and the heart, afford a striking illustration of the additional organs, which have been given to the most perfect animals. These organs do not, in the least, directly contribute towards the production or elaboration of nutritious matter; and, therefore, they may with propriety be regarded as of secondary, and not of primary importance. The different excretions remove a variety of substances unessential to the system, which, if retained, would disorder those functions that create, distribute, and assimilate the nourishing properties of food. Most of the excretory organs have been added to the more perfect forms of organization, for purposes of a secondary nature, in regard to the production of the pabulum vitæ, but of vital consequence with respect to the continuance of life, from the very intimate relations existing between them and other organs which produce excrementitious matter." (P. 51.)

And, again, he observes:

"From these cursory observations we are prepared to understand the functions of those organs which are called primary, namely, the stomach, the liver, the pancreas, aud the various glands that in any way contribute towards the elaboration of food. Although these organs are distinguished by different actions and properties, yet they are all equally labouring to produce one indispensable substance, namely, chyle. This fluid bears the same relation to the animal, that sap does to the vegetable system.

"It is by means of chyle, that the continual wastes of the frame are compensated; that every part of the organized being is more fully developed in the period that elapses between infancy and manhood; and that the various powers of life are invigorated and enabled to continue their operations, amidst extensive and constant changes, with unabated energy. But chyle is not calculated to answer any of these purposes, until it has passed through the lungs. The body can receive no nourishment whatever from any substance, unless this has undergone certain modifications in these organs. What the precise changes are, which are produced upon it, are unknown; but there is little doubt, that they are chemical and various in their nature. The ascending sap, until it has been acted upon by atmospheric air, is no more calculated to contribute to the nourishment and vigour of the tree, than chyle, before it has been exposed to the influence of the same renovating principle, is adapted to preserve and sustain animal life." (P. 54.)

The two following conclusions seem the most important, and the most in point, which the author draws from this preliminary part of his research:

" 5. That the animal system cannot derive nourishment from any substance, unless it passes through the lungs; and still further, that the extent and perfection of assimilation will, in a great measure, be regulated by the extent and perfection of the chemical changes which the blood undergoes in the respiratory organs.

" 6. It is also manifest, that there are no organs of the body, except the lungs, which can, under any circumstances, produce chemical changes in the circulating fluid; any more than the bare trunk is capable of effecting in the tree those similar changes, which in summer are produced by its numerous leaves. The lungs are, therefore, to be regarded as the only cause of animal heat and of those chemical changes which the blood undergoes in passing from venous to arterial." (P. 66.)

For on these depend his doctrine of "*the nature of fœtal life*," in which the first point sought to be established is the "*essential distinction between embryotic and fœtal life*;" a distinction truly essential for the purpose of this essay, as the author's explication of the mode in which the fœtus is nourished will be found wholly inapplicable to the explication of the nourishment of the embryo. It is truly said,

"Those, who endeavoured to shew that it is entirely supported by the maternal blood, found a difficulty in accounting for its nourishment and growth during the time previous to a connexion being established between the uterus and the chorion; others, who advanced a different opinion, were inclined to think that the fœtus is altogether indebted for its nutrition to the fluids in which it is immersed. In support of the latter supposition it is stated, that these exist at a period when it cannot possibly have other means of sustenance." (P. 67.)

"The ovum," it is argued, "which is found in the uterus immediately after impregnation, possesses within itself not only the principles of vitality, but fluids which are appropriated to the purposes of organization;" that a vascular connexion with the uterus would be of no service to the embryo before the internal organs are sufficiently developed to receive and distribute blood; that the ovum is, as it were, partly hatched by the warmth of the uterus; and that

"When the vital actions have proceeded so far in the organization of the fœtus, as to create, although imperfectly, the heart, the vena porta and the aorta, the umbilical cord may be distinctly perceived, and it very soon establishes a connexion between the chorion and the uterus. When this union is fully established the embryotic is changed into fœtal life, and the latter is exclusively nourished by the arterial blood of the mother." (P. 69.)

Here it may not be amiss to note, that in kangaroos, opossums, and other marsupiate animals, there is no umbilical cord, no connexion of the fœtus with the uterus at any time; and although birth takes place at a very early period of gestation, still the young are born in their fœtal, not their embryotic states.*

From this first position, the author proceeds to establish his second, which relies on the conclusions already quoted, viz.

"That whatever supports the animal system necessarily undergoes a similar process in the lungs before it becomes nutritious, and that this process is always accompanied by a variety of excretions commensurate in quantity with the action of those organs that produce chyle. It is therefore evident, that the fœtus is not nourished by the amniotic fluid: if it were, the liver, the pancreas, and the stomach would be almost as vigorously exercised, as they are immediately after birth, when the intestines, kidneys, lungs, and skin, most actively perform their several offices. The fœtus cannot possibly breathe by the mouth, neither does it evacuate fœcal matter to permit successive accumulations of it in the bowels, which are not burdened by any excrementitious matter.

"If we even allow that the amnios, from its great purity, does not require to be considerably changed in order to be converted into chyle, it is nevertheless true, that the modifications it undergoes,

* Dr. Granville, in his very interesting work "St. Petersburgh," vol. i. p. 233, states that he saw, in the museum of Dr. Froriep, at Weimar, a preparation of "a fœtus, ten weeks old and well proportioned, without the slightest indication of a cord, or of the usual mark of its insertion." Dr. G. adds, that he knows only of two other examples of this rare aberration of nature; the one at Ghent, the other at Göttingen. Dr. Mason Good published, many years ago, an account of a remarkable case of Preternatural Fœtation: it was a case of twins; the first child was born alive. It had no sexual characteristic; neither penis nor pudendum; "it had neither anus, funis, nor umbilicus." The other child was perfect in every respect. Dr. Good considered the liquor amnii to be the proper source of nourishment for the fœtus. For the detailed description of this very singular case, we refer the reader to our Journal for February 1829, p. 166.—Editor.

however slight they may be, would, during the period of fœtal life, create an abundance of the different excretions. The meconium which is found in the intestines does not possess the peculiar qualities of alvine evacuations. That the amnios serves the purposes of nutrition, is an idea that cannot for a moment be entertained, when we consider the changes to which chyle must, according to the statements already made, be necessarily exposed in the lungs before it can acquire the properties of aliment. From the principles previously stated, it appears unreasonable to suppose that assimilating operations are continually going on, unless it be also allowed, that they are invariably accompanied by a proportionate exercise of the excretory organs." (P. 74.)

Among other objections to the doctrine that the fœtus is nourished by swallowing and digesting the liquor amnii, it is stated "that a person destitute of sensorial sensibility (and *the fœtus has none,)* cannot exercise the faculty of deglutition:" whereas, on the contrary, Dr. Blundell asserts that he, on inserting his finger into the mouth of the fœtus, during the operation of turning, found, in two instances, that it sucked as vigorously before birth as afterwards; thus shewing (as he argues) that it felt hunger: moreover, that it perceived the finger, and that it had sense enough to perform the operation of sucking; and, therefore, that its mind was in action." We do not believe that the fœtus does swallow the liquor amnii; still we do not think that facts bear out the assertion of its insensibility, so decidedly advanced in the following passage:

"The nutrition of the fœtus cannot certainly be influenced by impressions communicated to the brain, because, in it, this organ is insensible to them. Motion is displayed by the fœtus, which might lead the hasty observer to suppose that it demonstrates the sensibility of the brain; but this phenomenon is as strongly manifested when the brain is wanting, or defective, as when it is quite perfect. If hunger were felt by the fœtus, it would most probably cause an action in the organs of deglutition; but as the desire of food is a sensation of the mind, and indicates, like the faculty of reflection or attention, a wakeful state of the sensorium, it cannot with propriety be said to exist at this time." (P. 78.)

The next subject discussed is the power attributed to the placenta, of oxygenating the blood it receives; which hypothesis is declared to be absurd, for it is stated,

"As a universal law, that whenever venous becomes arterial blood, air is received directly from without, and whatever is excrementitious is expelled directly from within." (P. 85.)

And in the chapter on "*the functions of the Placenta,*" the author's views seem fully embodied in the four following propositions:

"1st. The fœtus derives its blood exclusively from the mother.

"2dly. The placenta is an organ incapable of producing chemical changes in the blood.

"3dly. The fœtus has not in activity any organs than can in the least oxygenate the blood it receives.

"4thly. Venous blood cannot support organic life." (P. 94.)

Having disposed of the negative, we are now at liberty to examine our author's positive affirmations, which commence with the anatomical enunciation that the uterus "is supplied with four arteries, two *spermatic*, and two *uterine*:" from which circumstance it is argued that

"It is scarcely possible to conceive that the office of these vessels is merely to support the vitality of the organ in its unimpregnated state, because, at this time, it is extremely diminutive, and consequently requires only a small portion of blood. It appears rational to imagine that they are formed principally to convey to the fœtus an arterial fluid. These vessels are enlarged, and are likewise rendered more active, as the fœtus advances towards maturity. Such changes are what the physiologist would anticipate from a knowledge of the dependence of the fœtus on the maternal system for its support." (P. 103.)

"That the embryo does not depend on any mysterious adaptations of the uterus, is proved by its growth in cases of extra-uterine developement. The causes of its production in the uterus occasion its formation in every unnatural situation. If the fœtus is connected with the parietes of the intestines, which it sometimes happens to be, it continues to grow, until the general derangement it occasions in the maternal system, destroys its own being, or that of the mother. It has formed an adhesion to these parts, from the stimulus imparted to their vessels, by the manner in which it acts on those of the internal surface of the uterus. The principal difference between this organ, and the different viscera, to which it is sometimes attached, is in the quantity of blood furnished for its nutrition. When the fœtus is extra-uterine, it is sometimes capable of existing only a short time, because it does not receive blood adequate to its necessities. The small arteries belonging to the intestines are large enough to maintain their vitality and functions; but when a small portion only of their surface is occupied by a placenta, an abundant supply of arterial blood cannot be afforded to the fœtus. During pregnancy, the uterus becomes more vascular than any other part of the system under similar circumstances, and it is, therefore, well adapted to nourish the fœtus in every stage of its progress. I cannot conceive any other mode of explaining, in a satisfactory way, the manner in which a connexion is formed between the embryo and uterus, or any other part of the system. The blood of the mother must necessarily pass from the uterine vessels to the placenta, which it could not regularly do, unless the relations between the two organs were such as I have described.

" The explanation which I have here given is materially strengthened by the fact related in the following passage from Magendie: " In bitches, about the middle of their gestation, there are seen a great number of small arteries, which, issuing from the tissue of the uterus, pass into the placenta, where they are divided into several ramifications. At this period, it is impossible to separate these two organs, without tearing these arteries, and producing a considerable hemorrhage." (P. 106.)

" When a quantity of camphor" observes Majendie, " is injected into the veins of a dog, the blood soon takes a strong odour of camphor. After having made this injection into a bitch with pups, I extracted a fœtus from the uterus; at the end of three or four minutes its blood had no odour of camphor; only a second fœtus, extracted after a quarter of an hour, had a strong odour of camphor. It was the same with the other fœtuses." (*Appendix*, p. 1.)

" The uterus does not become disorganized from its various modifications during gestation, nor the organ to which it is attached, but, on the contrary, blood continues to flow indirectly, from the mother to the fœtus, and in the same way from the fœtus to the mother, without any interruption to its circulation. These peculiar circumstances can scarcely be supposed to exist in diseases: 'The liquors,' as Monro, Primus, justly observes, 'are not carried from the mother to the fœtus, or from the fœtus to the mother by continued canals, that is, the uterine arteries and veins of the secundies; but the extremities of the umbilical vein take up the liquor by absorption, in the same way as the lacteals do in the guts; and the umbilical arteries pour their liquors into large cavities of the sinuses or other cavities analogous to them.'

" If this view be correct, we cannot but observe the wisdom which nature has shewn in the construction of the placenta. If we suppose the connexion between the mother and placenta to be maintained by fifty, or any number of vessels; a mode of union which is conceived to exist by several distinguished physiologists, it will necessarily follow, that whenever physical or moral causes disturb the general circulation of the mother, that of the fœtus will be disordered to an equal extent, and consequently its existence rendered extremely precarious.

" It is well known that the maternal system is frequently subject to severe and sudden disturbances. If we imagine a continuous stream of blood flowing from the mother to the fœtus, it is evident that the latter will be affected in proportion to the excitement or depression of the circulatory system of the former: and if such were really the nature of the connexion between them, I cannot conceive how the life of the fœtus could be preserved when the maternal system is particularly disturbed. But, if the placenta be regarded as an organ intervening between the mother and the fœtus, for the purpose of receiving the blood of both, it is not difficult to understand how a temporary derangement of the maternal circulation seldom materially affects that of the fœtus. The placenta is therefore a barrier to the transmission of injurious effects, arising from sudden and violent emotions of mind, or from a variety of other causes that render irregular the general circulation of blood." (P. 109.)

From these extracts, it is evident that our author considers the placenta to be like the spleen, a diverticulum, by which the circulation is regulated from the mother to the fœtus, for the actual existence of which he stoutly argues; and the contrary opinion, although supported by some few no inconsiderable authorities, he declares an impossibility, and denominates *absurd*, (vide pages 114, 115, &c.;) and this subject is concluded with some hints as to the cause of the separation of the placenta from the uterus after the expulsion of the child.

The sixth chapter is chiefly argumentative, and dedicated to an inquiry into the "*origin of the Liquor Amnii and Meconium:*" the first of which is denied to be a secretion from the amnios, and contended to be an excretion from the skin. One fact is, however, overlooked, which is somewhat important to the general views of the author, who states that, for the secretions and excretions to take place, there must be a constant supply of arterial blood; and yet the liquor amnii is found before the embryo has any attachment to the uterus. Moreover, it should not fail to be remembered, that the liquor amnii is present in those abortive ovula which do not contain a fœtus, and in which there is no umbilical cord to convey blood from the uterus to its contents. And, as to the latter, he concludes that the meconium appears, then, to be nothing more than the admixture of the different secretions of the abdominal viscera, and is not, in any degree, the result of a regular digestive process.

Having thus at length "endeavoured to prove that the nourishment of the fœtus is derived exclusively from the arterial blood of the mother," it becomes necessary to prove what "has already been stated in the previous pages, viz. that the umbilical vein carries arterial blood from the placenta to the fœtus;" and for this purpose the following experiments are given.

"This opinion is supported by Drs. Bostock and Jeffrey. Physiologists in general are either opposed to this conclusion, or consider it doubtful. It is really astonishing, that a subject so easy to investigate, and to decide by experiment, has been allowed to remain so long undetermined. From the kindness of my friend Mr. Carr, surgeon, Sheffield, I have been enabled to prove, by an experiment of the simplest kind, that the umbilical vein circulates arterial blood. The following was the method employed by Dr. Jeffrey: He took part of the cord, and dissected away the gelatinous substance of it, until he had laid bare the vessels, when, on puncturing them, he observed that there was a difference of colour between the blood in the vein and the arteries. Many physiologists are said to have failed in this experiment. From the tedious preparation which it required, I did not succeed to my full satisfaction. But on taking part of the cord, as soon as the child was born, around which I had previously tied a ligature, about two or three inches from the free extremity, and cutting this with a sharp scalpel, in order to make an even surface, I very clearly discerned, on pressing the cord from below upwards, blood of a very different colour, flowing from the umbilical vein and arteries. If the experiment does not fully succeed in the first instance, tie a ligature still farther removed from the end, and, having made a fresh surface, press gently from below upwards. Sometimes a large drop of florid blood is observed to stand directly over the umbilical vein, and another dark coloured over the arteries, without their being in the least mingled with each other, and, in this case, the difference between the two is so striking that no one can fail to observe it." (P. 152.)

The result of these experiments are, however, it cannot fail to be remarked, diametrically opposite to the observations of other physiologists, who state that there is no perceptible difference in the colours of the blood in the umbilical arteries and vein. Dr. Blundell states, that when he made experiments to determine the point, that he was "unable to discover any manifest difference in the colour of the two: if difference existed at all, it consisted in a mere shade;" and, in Elliotson's translation of Blumenbach's Physiology, we find the following note, which declares not only that "credible authors have asserted that the eye cannot distinguish between the arterial and venous blood of the fœtus," but also that Bichat could observe no difference in the arterial and venous blood of the umbilical cords of several guineapigs examined, while the mother's respiration was still continuing after an opening had been made into the abdomen: "les deux sangs offroient une noirceur egale." *(Recherches Physiologiques.)* So, too, in regard to dogs. *(Comp. Anat.)*

The following calculation is interesting, as exhibiting the final cause of the more rapid circulation in the fœtus.

"It is an interesting and important inquiry whether the whole of the fœtal blood flows through the umbilical arteries to the placenta, in the same time as the whole of the sanguineous fluid of the mother circulates through the lungs, viz. in about three minutes. If such be the case, the fœtus will have the whole of its blood renewed as frequently as that of the maternal system. There is certainly the same relation between the heart of the fœtus and the quantity of blood belonging to its body, as there is between the heart of the adult and the quantity of blood circulating in its system. If this assertion be allowed to be correct, and it is almost impossible to deny its accuracy, we shall be able to estimate the difference between the properties of fœtal and maternal blood. At every contraction of the left ventricle of the heart in the adult, a certain quantity of blood is expelled, the whole of which must inevitably return to the right auricle and be sent to the lungs, before it can arrive at the point from which it departed. This revolution is supposed to be performed in about three minutes. The blood transmitted at every contraction of the left ventricle of the heart of the fœtus, does not return, in the same manner, to the right auricle: one portion is sent to the internal iliacs, and conveyed from them to the placenta, by means of the umbilical arteries; another pursues its regular course, circulating from arteries into veins, until it arrives at the right auricle, where it is blended with the arterial fluid derived from the umbilical vein which terminates in the vena porta and vena cava inferior. This peculiarity in the circulatory system of the fœtus appears calculated to produce blood of an inferior quality, and, indeed, the consideration of this circumstance, has induced Coleman, professor of veterinary surgery, very incorrectly, to call the fœtal blood black.

"The whole mass of the blood of an adult circulates throughout the system once every three minutes, because the heart contracts about seventy-five times per minute; if it contracted oftener with equal or superior energy, the same quantity of blood would complete its revolution in considerably less time. The heart of the fœtus, instead of contracting seventy-five times per minute, contracts, at least, 120 times. In considering this subject, we must not forget that there is the same relation between the heart of the fœtus and the quantity of blood belonging to its body, as there is between that of the adult and the quantity of blood circulating in its system.

"It is therefore obvious, if the fœtal heart contracts 120 times per minute, that the complete circulation of the blood in its system will be effected in almost half the time of that of the adult, and consequently, although venous blood is always mingling with the arterial fluid of the umbilical vein, the blood of the fœtus cannot be particularly deteriorated from this circumstance, because the whole mass is frequently renewed by the very numerous contractions of the heart. If this contracted seventy-five times instead of 120, the relation between it and the mass of the circulating fluid remaining the same, the arterial qualities of the blood would be diminished almost one half." (P. 154.)

Chapter VIII. is ushered in with the declaration that "the brain, the spinal cord, the stomach, the liver, the pancreas, and the intestines, are not essential to fœtal life;" for, in various cases of monstrosity, these parts may severally be absent, and yet the fœtus grow. Again,

"It must be allowed, that the object of the functions of the primary organs is to provide the system with arterial blood, and, this being granted, it is quite manifest that these functions are not essential to the fœtus, as it is supplied with arterial blood from another source." (P. 161.)

Furthermore, with regard to "the functions of the liver, the supra-renal capsules, the thymus, and the thyroid glands, in the fœtus," we read,

"It is well known that these organs are proportionally much larger in the fœtus than in the adult. This striking difference in their size is probably occasioned by the great dissimilarity in the circulation of the blood before and after birth. In the infant there is a natural and constant tendency to indulge in muscular exercise; its frequent cries, and those external objects which almost incessantly call forth the expression of its various feelings, produce one general effect, viz. a more equable distribution of blood." (P. 167.)

And again,

"We may, therefore, conclude, that the large size of the liver in the fœtus, arises from the great quantity of blood which it receives, and the inactivity of those causes which invigorate the general circulation. The supra-renal glands are also observed to be very large in the fœtus. These I am inclined to consider diverticula, operating more or less in every period of life, in order to secure the proper and regular action of the kidneys, which are liable, at times, to be disordered by irregularities in the circulation. Nature has, throughout the animal economy, protected the most essential organs from sudden destruction or derangement. The five senses have their appropriate means of preservation; the heart and the lungs are secure from external injury by the strong parietes of the chest, and from occasional congestions by the liver and the spleen: and other organs, as the kidneys and the larynx, have also auxiliary appendages, which render them less liable to disorder.

"There is a striking difference between the structure of those organs which directly support the system by their respective functions, and that of others, which I suppose to act wholly, or in part, as diverticula. The former are susceptible of increase of volume in one way only, by an accretion of growth of new matter; the latter are capable of great augmentation, without any permanent addition being made to their substance." (P. 170.)

In the xth and xith chapters, on "*the Mode of Nourishment in the Oviparous and the Ovo-viviparous Animals,*" and on "*the Influence of the Imagination of the Mother on the Development and Constitution of the Fœtus,*" although the titles would seem to promise much matter of interest, we do not find any thing worthy of particular note, either on account of its originality as a speculation, or its novelty as a fact; we shall, therefore, take our leave of a volume which we have read with no slight interest, although the dictatorial style in which many parts are written, and the continual digressions in which the author has indulged, have, it must be confessed, in some slight degree, alloyed the unmixed pleasure that we should have otherwise received.

### COLLECTANEA.

